# Spontaneous imbibition and oil displacement experimental investigation in fracture–matrix cores of tight sandstone reservoirs

**DOI:** 10.1038/s41598-026-44044-z

**Published:** 2026-03-18

**Authors:** Weihua Chen, Rui He, Li Li, Jiejing Bai, Zhengyong Li, Tao Wang, Le Luo, Xinyu Zhang, Wei Zhang

**Affiliations:** 1 Engineering Technology Research Institute of Southwest Oil & Gas Field Company, PetroChina, Chengdu, 610017 China; 2 Shale Gas Geological Evaluation and Efficient Development of Sichuan Provincial Key Laboratory, Chengdu, 610017 China; 3 Chuanzhong Oil and Gas District of Southwest Oil & Gas Field Company, PetroChina, Suining, 629000 China; 4 Development Division of Southwest Oil & Gas Field Company, PetroChina, Chengdu, 610051 China; 5 State Key Laboratory of Deep Oil and Gas, China University of Petroleum (East China), Qingdao, 266580 China

**Keywords:** Tight sandstone reservoirs, Fracture–matrix cores, Spontaneous imbibition, Oil displacement, Energy science and technology, Engineering, Materials science

## Abstract

Tight sandstone reservoirs are characterized by low porosity and low permeability, which results in great difficulty in oil production and low recovery. In this work, based on fracture–matrix tight sandstone core models and field crude oil, the interfacial activity and reservoir adaptability of the oil displacement agent C-22 were evaluated. Subsequently, oil displacement agents with different interfacial tension levels were optimized and selected as control groups for subsequent oil displacement experiments. The migration behavior of crude oil in fracture–matrix cores during spontaneous imbibition and oil displacement processes using C-22 was systematically investigated, and the key development parameters for oil displacement were further optimized. The results show that C-22 exhibits excellent interfacial activity and good reservoir adaptability. An ultralow interfacial tension of 0.12 mN/m can be achieved at a concentration of 0.1 wt%, and the interfacial tension remains stable after 7 d of aging. In the oil displacement experiments, as the pressure decreases from 15 to 0 MPa, the final oil recovery reaches 18.94%. Nuclear magnetic resonance analysis indicates that oil in mesopores and macropores is predominantly mobilized at the early stage, whereas oil in micropores is mainly produced at the later stage. Furthermore, the key development parameters for the oil displacement process were optimized. The optimal oil displacement performance is achieved when the interfacial tension is reduced to the 10^−2^ mN/m level, the concentration of C-22 is 0.2 wt%, and the shut-in time is 12 h. We expect that this study can provide valuable insights into the effective development of tight sandstone reservoirs and offer theoretical guidance for the selection of field operational parameters.

## Introduction

The global share of unconventional oil and gas resources is steadily increasing. As most conventional reservoirs have entered the late stage of development, unconventional resources have become a critical focus for exploration and production^1–3^. Tight sandstone reservoirs, as representative unconventional reservoirs, are characterized by poor petrophysical properties, low permeability, pronounced fracture–matrix heterogeneity, complex pore structures, and strong anisotropy^4–6^. These features lead to the limited effectiveness of conventional water injection in such reservoirs and place greater demands on advanced reservoir stimulation technologies, such as hydraulic fracturing^7,8^.

Hydraulic fracturing is a key stimulation technology for the development of unconventional oil and gas reservoirs^9,10^. It enhances well productivity by injecting high-pressure fracturing fluids to generate artificial fractures, thereby improving the permeability of hydrocarbon-bearing formations. However, the narrow pore throats and limited flow capacity inherent to tight sandstone reservoirs restrict the overall effectiveness of stimulation^11–13^. Therefore, to achieve efficient reservoir modification, it is necessary that, following the creation of a large-scale fracture network by hydraulic fracturing to enhance crude oil mobility, the fracturing fluid continues to function as an effective agent for imbibition and displacement^14–16^. This ensures that the energy invested in fracturing is utilized to the maximum extent possible.

The integrated fracturing flooding technology has been proposed and has attracted widespread attention^17,18^. This approach involves a shut-in period after the fracturing fluid completes fracture creation, during which imbibition and displacement occur between the fracturing fluid and crude oil. Driven by the pressure differential during flowback, well productivity can be significantly improved^19,20^. Imbibition refers to the process in which the wetting phase displaces the non-wetting phase under the influence of capillary forces in the porous medium^21–23^. Since the nineteenth century, extensive studies have been conducted worldwide to investigate the role of imbibition in enhancing oil recovery^24–26^. Yang et al.^27^ examined the influence of fracture distribution on surfactant imbibition in tight sandstone reservoirs and found that fractures can effectively reduce oil droplet adsorption on the core surface, increasing imbibition recovery by approximately 10%. Moreover, surfactants were shown to further improve oil recovery by about 15% through enhancing water wettability of the rock surface, reducing interfacial tension, and weakening oil droplet adhesion. Liu et al.^28^ developed a novel hydrophilic silica, and spontaneous imbibition experiments demonstrated that it can readily enter reservoir pores and adsorb onto rock surfaces, alter wettability to promote crude oil detachment, and ultimately achieve an oil recovery of 36%. Despite the extensive research on surfactant flooding and spontaneous imbibition, studies that specifically elucidate oil-phase flow characteristics and mobilization mechanisms in fracture–matrix core systems remain limited. During production, several key challenges must be addressed, including the coupling between interfacial tension and capillary forces, preferential channeling of displacing fluids along fractures, coupled fracture–matrix flow, low sweep efficiency, and difficulties in pore–throat characterization and large-scale numerical simulation^29,30^. Therefore, there is an urgent need to clarify the oil flow behavior in both fractures and the matrix and to elucidate the mechanisms governing oil mobilization within pores during spontaneous imbibition.

In this work, fracture–matrix tight sandstone core models and field crude oil were employed to evaluate the interfacial activity and reservoir adaptability of the oil displacement agent C-22. Oil displacement agents with different interfacial tension levels were subsequently optimized and selected as control groups for comparative oil displacement experiments. The migration behavior of crude oil in fracture–matrix cores during spontaneous imbibition and displacement processes using C-22 was systematically investigated, and the key operational parameters governing oil displacement were further optimized. Based on these results, an oil displacement strategy tailored for tight sandstone reservoir is proposed, providing theoretical insight and technical support for the efficient development of tight sandstone reservoirs.

## Experimental component

### Experimental materials

Materials: Crude oil, sourced from Well X located at a tight oil field in Western China; Oil displacement agent C-22, sourced from the laboratory of the tight oil field; Lauramidopropyl betaine (LHSB), sourced from Shanghai Aladdin Biochemical Technology Co., Ltd.; Tetradecyl hydroxypropyl sulfobetaine (THSB35), sourced from Shanghai Aladdin Biochemical Technology Co., Ltd.; Cocamidopropyl betaine (CAB-35), sourced from Shanghai Deyi Chemical Co., Ltd.; Sodium dodecylbenzenesulfonate (LAS-30), sourced from Linyi Lvsen Chemical Co., Ltd.; Alcohol ethoxylate 15 (AEO15), sourced from Shanghai Aladdin Biochemical Technology Co., Ltd.; Sodium nonylphenol ethoxylate (10) sulfate (NPES), sourced from Jiangsu Haian Petrochemical Plant; Heavy water, sourced from Shanghai Aladdin Biochemical Technology Co., Ltd.; Sandstone core, sourced from Well X.

### Apparatus

Equipment: PS-80 A CNC Ultrasonic Cleaner, Dongguan Jiekang Ultrasonic Equipment Co., Ltd.; Multi-functional Flow Test and Evaluation System, Beijing Yongruida Technology Co., Ltd.; MacroMR12-150 H-I Online Nuclear Magnetic Resonance Analysis and Detection System, Suzhou Niumag Analytical Instrument Co., Ltd.; Analytical Balance, Mettler-Toledo International Trading (Shanghai) Co., Ltd.; UPR Series Ultra-Pure Water System, Sichuan UPR Ultra-Pure Technology Co., Ltd.; Vacuum Drying Oven, Qingdao Lanten Science and Education Instrument Equipment Co., Ltd.; TX-500 C Interfacial Tensiometer, Krüss GmbH, Germany; High-Pressure Oil Saturation Device, Haian Petroleum Scientific Research Instrument Co., Ltd.

## Methods

### Determination of the amount of oil displacement agent

1. All syringes, sample tubes, and tube caps were cleaned with petroleum ether and ethanol, followed by rinsing with the test solution.
2. After rinsing, an appropriate volume of the test surfactant was drawn into a syringe and slowly injected into the sample tube, ensuring that no air bubbles were generated.
3. An appropriate amount of the test formation oil was then drawn into another syringe, and a single droplet was injected into the sample tube. The syringe was quickly withdrawn to ensure the droplet remained suspended without adhering to the tube wall.
4. The sample tube was held horizontally, securely capped, and placed onto the instrument’s rotating shaft, after which the shaft cap was attached. The interfacial tensiometer and associated software were turned on, and the temperature was set to 68 °C, the rotational speed to 6000 r·min⁻¹, and the density difference between the oil sample and the test solution was input. The test was then initiated.
5. The microscope was fine-tuned to locate the target oil droplet. The leveling button was used to keep the droplet stationary within the on-screen field of view. After stabilization, the interfacial tension was measured.

### Static spontaneous imbibition and T_2_ spectrum experiments

1. The constant-temperature water bath was switched on and set to 68 °C.
2. The imbibition cell was cleaned sequentially with petroleum ether, ethanol, and the test fluid.
3. The core was placed at the bottom of the imbibition cell, which was partially filled with the test fluid without exceeding its rim. Vaseline was applied around the rim, the cell was covered with its lid, and the seal was reinforced with plastic film to prevent evaporation.
4. The test fluid was continuously added *via* the extended rubber tubing until the imbibition cell was completely filled.
5. The volume of expelled oil was recorded at designated time intervals, and the recovery was calculated accordingly.
6. The *T*_2_ spectra were measured using the Nuclear Magnetic Resonance (NMR) system at 0 h, 48 h, 96 h, and 144 h of the imbibition process.

### Displacement experiment

1. The core was prepared into a fractured core using a core orientation and splitting device. Following cleaning and drying, the core was saturated with crude oil using a vacuum-pressure saturation system.
2. The multifunctional flow test and evaluation system was started. The sample solution was filled into the intermediate container, the ISCO pump was activated, and the core was placed into the core holder with all flow lines connected. The oven temperature was set to 68 °C. After temperature stabilization, the confining pressure of the core holder was set to 18 MPa and the back pressure to 15 MPa.
3. The valve at the outlet end of the core holder was closed. The ISCO pump was reactivated and set to constant pressure mode. The sample solution was injected into the crude oil-saturated fractured core from the inlet end until the system pressure stabilized at 15 MPa. Subsequently, the inlet valve was closed, and the shut-in period was initiated.
4. Depletion production was simulated by gradually reducing the pressure at the outlet end using the back pressure regulator, with stepwise decreases to 10 MPa, 5 MPa, and finally 0 MPa. Pressure changes and recoveries were recorded throughout the entire process.

### Nuclear magnetic resonance experiment

Based on the aforementioned oil displacement experimental procedure, the online nuclear magnetic resonance analysis and detection system was employed to scan the *T*_2_ spectra of the fracture–matrix core saturated with simulated oil under initial conditions and at pressure reduction stages of 10 MPa, 5 MPa, and 0 MPa, respectively. The distribution of remaining oil within the core was observed, and the extent of oil phase mobilization in the core matrix was analyzed. Throughout the NMR scanning experiment, heavy water was used to prepare all solutions in order to eliminate interference from water signals^31,32^. The parameters of the cores used in the experiment are listed in Table 1.

**Table 1:** Core parameters.

Number	Length/cm	Diameter/cm	Porosity/%	Permeability/10^−3^ µm^2^
1	5.06	2.48	4.98	0.13
2	4.96	2.47	4.87	0.15
3	5.04	2.49	4.76	0.12
4	5.04	2.48	4.80	0.13
5	5.08	2.49	4.86	0.17
6	5.03	2.49	4.93	0.12
7	4.99	2.50	4.95	0.13
8	4.99	2.50	4.95	0.13
9	5.01	2.53	4.94	0.17
10	5.03	2.52	4.98	0.12
11	4.98	2.47	4.81	0.12
12	5.02	2.51	5.01	0.16
13	4.97	2.47	4.96	0.15
14	4.98	2.50	4.79	0.14
15	5.00	2.48	5.02	0.17
16	5.01	2.47	4.84	0.16
17	4.99	2.52	4.78	0.18
18	5.03	2.52	4.89	0.13

## Results and discussion

### SARA analysis, density, and viscosity of field crude oil

The crude oil was analyzed for SARA composition, density, and viscosity, and the results are presented in Table 2. It can be seen that the content of saturated hydrocarbon and aromatic hydrocarbon in the light component of crude oil is more than 90%, which makes the crude oil show low density and low viscosity. This kind of crude oil has good fluidity and is easy to exploit.

**Table 2:** Crude oil composition and property analysis.

SARS analysis/%				Density/ (g/cm^3^)	Viscosity/mPa s
Saturates	Aromatics	Resins	Asphaltenes		
72.32	18.04	3.98	5.66	0.84	11.47

### Interfacial activity of oil displacement agents

#### Screening of control groups with oil displacement agents at different interfacial tension magnitudes

The core of the oil increasing effect of the working fluid for fracture–flooding depends on three factors: interfacial activity, wetting change performance and emulsifying solubilization performance. The interfacial activity can reduce the oil-water interfacial tension and weaken the adhesion of crude oil. The wettability change performance can change the wettability of rock from lipophilic to hydrophilic, and improve the efficiency of oil washing. The emulsification solubilization performance can make the residual oil form a stable emulsion and expand the displacement range. Surfactant is a commonly used oil displacement chemical agent because of its amphiphilicity. It can not only change the wettability of rock and reduce the interfacial tension of oil and water, but also regulate the above three properties, synergistically improve the effect of pressure flooding and increase oil production, and adapt to the development needs of different reservoirs. To meet the requirements of subsequent imbibition experiments, three surfactants with different magnitudes of interfacial tension were selected for comparison using a TX-500 C full-range spinning drop interfacial tensiometer based on reservoir crude oil. The screening results are presented in Table 3.

**Table 3:** Screening surfactants corresponding to crude oil with different orders of magnitude of interfacial tension.

System name	Concentration/wt%	Interfacial tension/(mN/m)
C-22	0.3	0.0599
LHSB	0.1	2.5580
THSB35	0.1	0.7923
CAB-35	0.1	1.0561
LAS-30	0.1	0.4044
AEO15	0.1	1.6145
NPES	0.1	1.0473
CAB-35: LAS-30 = 8:2	0.1	0.2219
THSB35: LAS-30 = 5:5	0.1	0.1436
THSB35: LAS-30 = 4:6	0.1	0.0184
THSB35: LAS-30 = 3:7	0.1	0.0068

Based on the experimental data, the following surfactants and their corresponding interfacial tensile strength levels were selected: THSB35 at a concentration of 0.1 wt% as the surfactant with interfacial tension at the 10^−1^ mN/m magnitude; C-22 at a concentration of 0.3 wt% as the surfactant with interfacial tension at the 10^−2^ mN/m magnitude. The blended system THSB35:LAS-30 = 3:7 as the surfactant with interfacial tension at the 10^−3^ mN/m magnitude.

#### Interfacial tension of oil displacement agent (C-22) at different concentrations

**Fig. 1:**
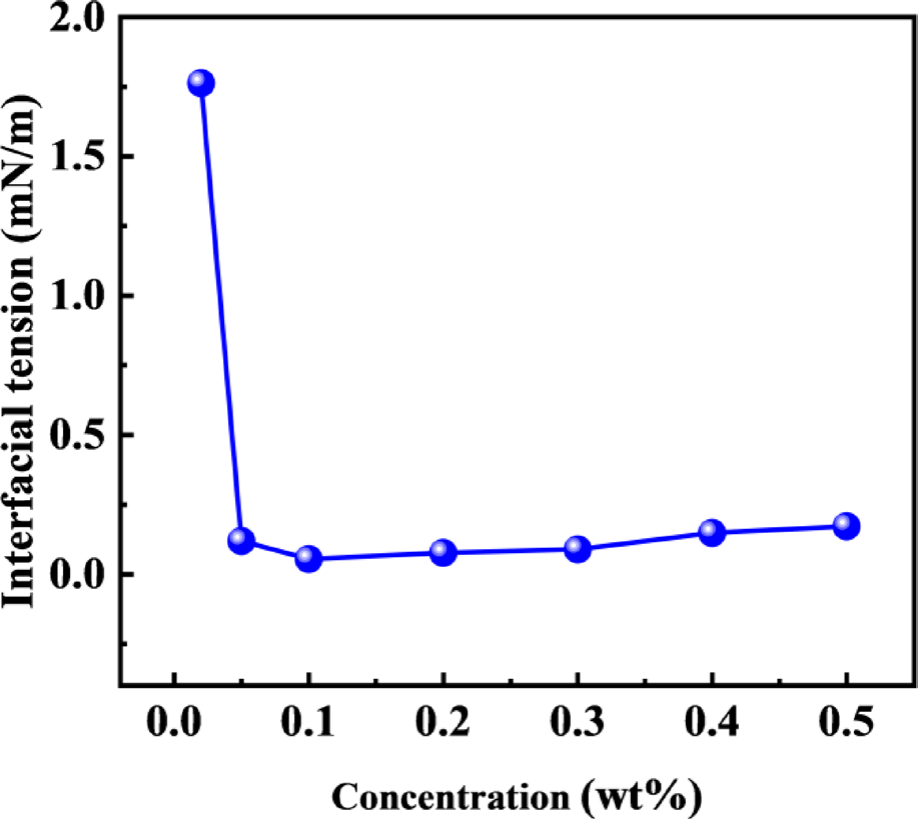
Interfacial tension between crude oil and oil displacement agents with different concentrations.

Interfacial tension is one of the key indicators for evaluating the performance of a oil displacement agent. A lower value signifies a stronger ability of the oil displacement agent to reduce oil-water interfacial tension and mobilize residual oil, thereby leading to higher displacement efficiency. To further understand the performance of the selected oil displacement agent (C-22), this study measured its interfacial tension against the target reservoir crude oil under different concentration conditions. The experimental results are shown in Fig. 1. The interfacial tension initially decreases, then stabilizes as the concentration increases. At lower concentrations, increasing the C-22 concentration allows more molecules to adsorb at the oil-water interface, effectively reducing the tension. When the concentration is sufficiently high and the interface becomes saturated, further increases in concentration primarily lead to the formation of micelles within the bulk solution, and no significant change in interfacial tension is observed thereafter.

#### Interfacial tension of oil displacement agent (C-22) at different aging times

In the process of fracturing-flooding, surfactant is used as the core oil displacement agent. After being injected into the subsurface, surfactants must endure formation high-temperature and high-pressure conditions, and their stability is a key performance indicator determining displacement efficiency^33,34^. Therefore, to evaluate the long-term stability of the oil displacement agent under simulated high-temperature reservoir conditions, the oil displacement agent solution was aged at reservoir temperature to mimic formation conditions. During the experiment, regular testing is required to observe whether the solution has state changes such as stratification and precipitation, and to monitor the fluctuation of interfacial tension, so as to provide key data support for the selection of oil displacement agents and the optimization of oil displacement schemes.

**Fig. 2:**
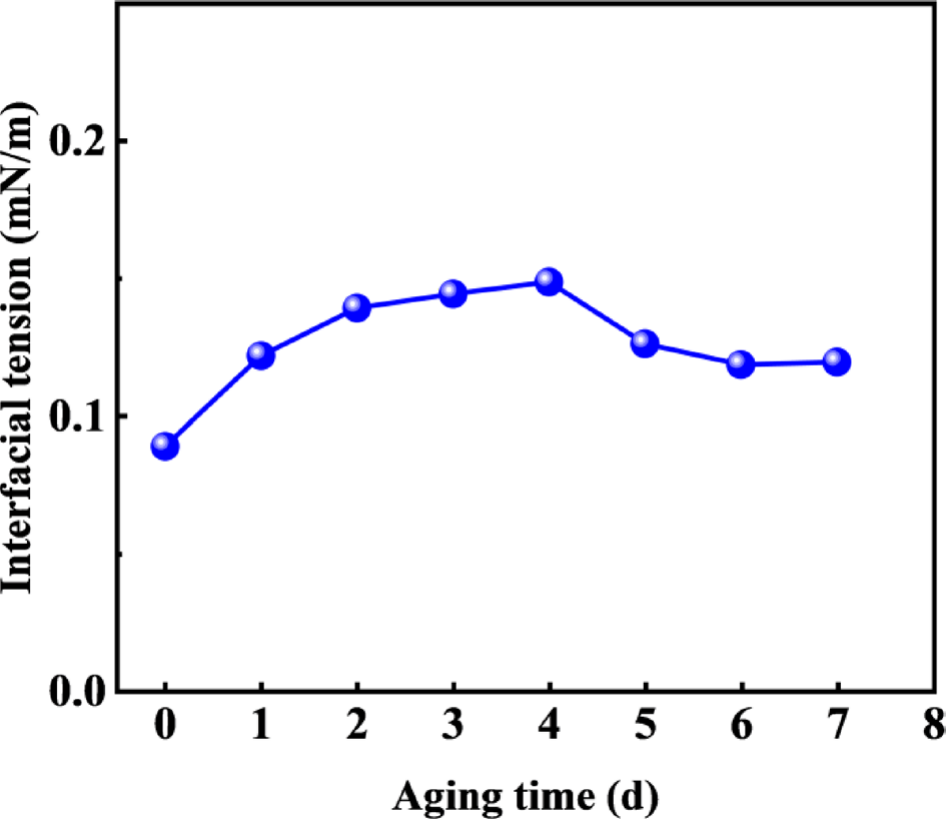
Variation of interfacial tension with aging time.

**Fig. 3:**
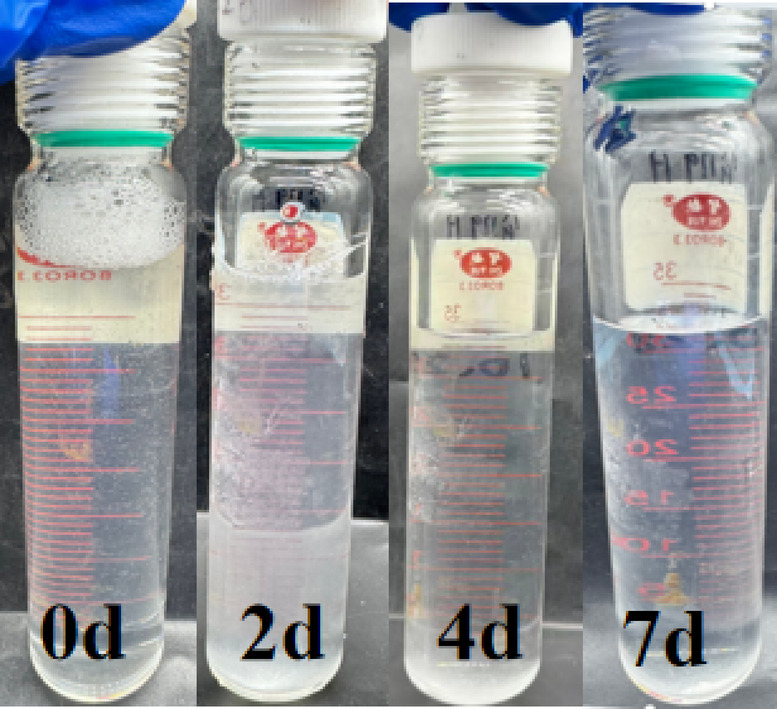
Variation of oil displacement agent solution with aging time.

Figure 3 shows photographs of the oil displacement agent solution aged at 68 °C during the aging process for 0, 2, 4, and 7 d, respectively. The solution remained clear and transparent throughout the 7 d aging period, preliminarily indicating its stability under simulated reservoir conditions. Interfacial tension measurements between the oil displacement agent and crude oil from the corresponding reservoir were conducted, with results shown in Fig. 2. The interfacial tension remained essentially stable as aging time increased, demonstrating that the oil displacement agent C-22 possesses good reservoir compatibility and can maintain effective oil displacement performance over extended durations.

### Spontaneous imbibition in fracture–matrix models

#### Evaluation of spontaneous imbibition recovery using oil displacement agents at different concentrations

Spontaneous imbibition occurs during the soaking process after the formation fracturing is completed. At this time, the working fluid for fracture–flooding encapsulates the rock matrix and is in a static state. At this time, the capillary force is the main force. The wettability of the imbibition medium determines whether the capillary force is the driving force or the resistance. Good emulsification disperses large pieces of crude oil into small oil droplets that are easier to pass through the pore throat of the rock. The imbibition medium enters the small pore channel to replace the crude oil into the large pore channel, and finally discharges the matrix system into the fracture or the wellbore. To investigate the effect of interfacial tension on crude oil flow during spontaneous imbibition experiments, tests were designed and conducted using oil displacement agents at different concentrations, corresponding to varying magnitudes of interfacial tension.

**Fig. 4:**
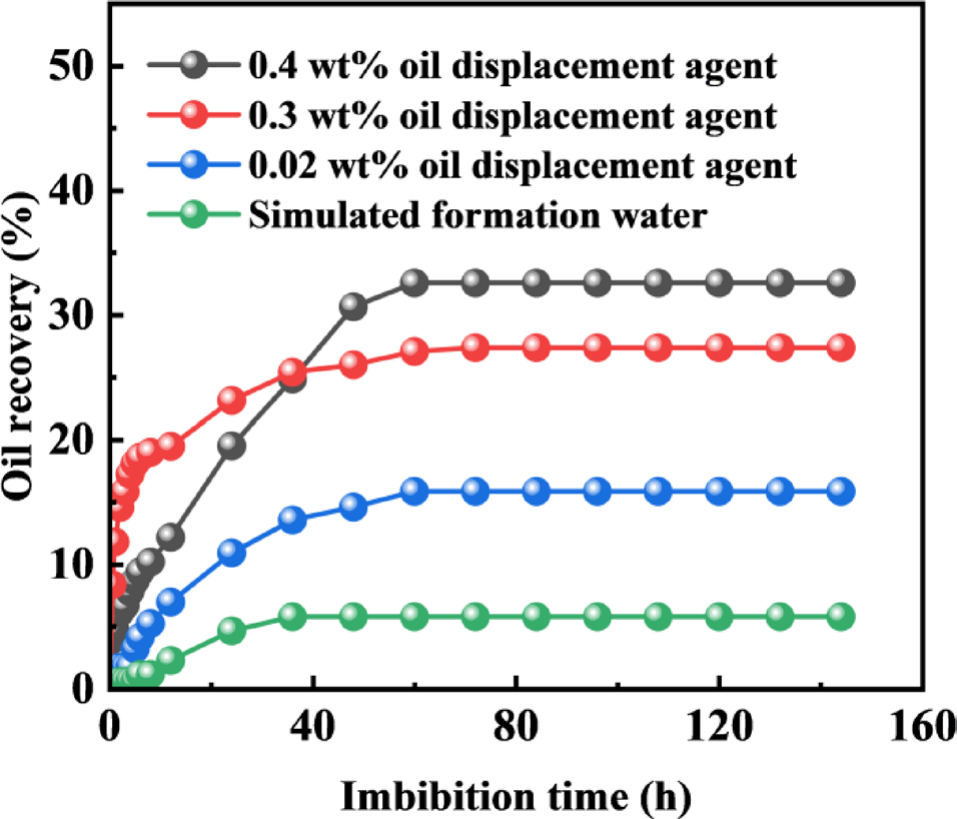
Variation of crude oil recovery via spontaneous imbibition versus time.

The spontaneous imbibition recovery results are shown in Fig. 4. The recoveries for simulated formation water, 0.02 wt%, 0.3 wt%, and 0.4 wt% oil displacement agent solutions were 5.79%, 15.87%, 27.37%, and 32.57%, respectively. As the concentration of the oil displacement agent C-22 increased, the imbibition recovery showed a progressive upward trend. Particularly in the early stage of imbibition, higher concentrations led to faster imbibition rates. At a concentration of 0.02 wt%, the interfacial tension was 1.76 mN/m. When the concentration of C-22 reached 0.3 wt%, the interfacial tension was 0.09 mN/m, resulting in a significant improvement in recovery. At a concentration of 0.4 wt%, the interfacial tension reached 0.15 mN/m. The oil displacement agent C-22 can more effectively reduce the oil-water interfacial tension, significantly lowering the flow resistance of crude oil in small pores and throats, promoting the emulsification and stripping of crude oil, and reducing its migration resistance. This enhances the liquid’s penetration ability within the rock pores, thereby promoting crude oil stripping and flow and improving the recovery. The highest imbibition recovery was achieved when the interfacial tension was on the order of 10^−1^ mN/m.

#### Characteristics of crude oil flow during imbibition in fracture–matrix cores

NMR technology can detect the signal of *T*_2_ relaxation of hydrogen nuclei, which effectively indicates the existence of substances that can produce this signal. Therefore, by using heavy water to shield the hydrogen signal of water in the oil displacement agent solution, the nuclear magnetic resonance *T*_2_ spectrum can only identify the hydrogen signal of crude oil in the core, so that the dynamic migration characteristics of crude oil in the large, medium and micropore throat system of the core can be qualitatively identified by this method, and the start-up, flow and retention mode of crude oil in the matrix can be clearly analyzed, which provides reliable experimental data support for optimizing the displacement scheme and enhancing oil recovery.

**Fig. 5:**
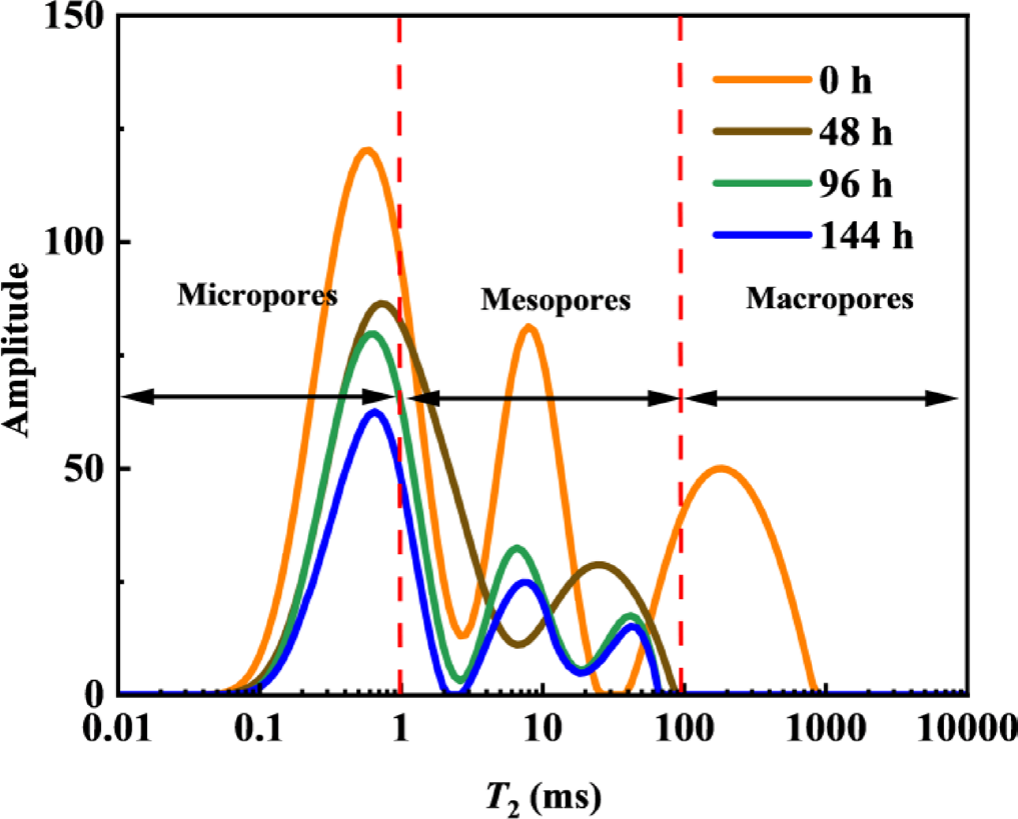
*T*_2_ spectra of crude oil during spontaneous imbibition.

As shown in Fig. 5, before the imbibition experiment began, the core saturated with simulated oil exhibited the highest signal intensity. As imbibition progressed, the NMR signal from within the pores continuously decreased. This indicates that crude oil in pores of different sizes was mobilized to varying degrees throughout the imbibition process. In the early stage of imbibition, the core signal changed noticeably. By 48 h of imbibition, the signal amplitude had decreased significantly, indicating that a substantial amount of oil had been displaced from the core. When imbibition continued beyond 96 h, the signal amplitude in the core still decreased but no longer markedly, suggesting that the oil-water migration within the core had largely stabilized.

**Fig. 6:**
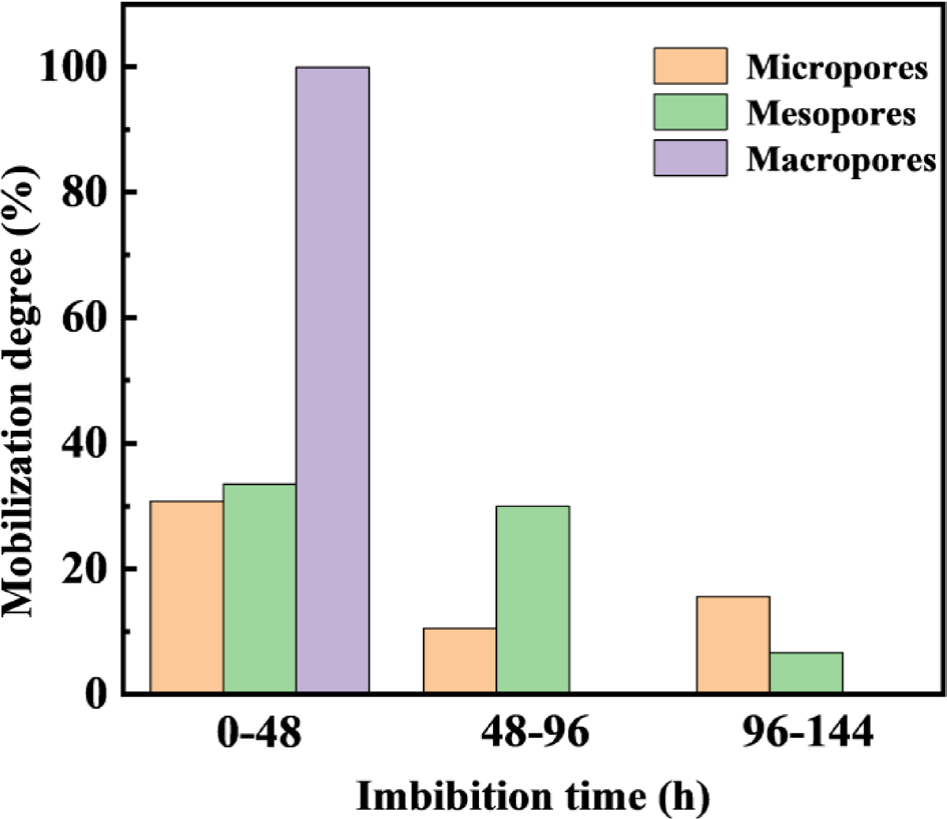
Mobilization patterns of crude oil in pores during spontaneous imbibition.

To further investigate the oil mobilization degree across different pore types during various imbibition stages, the area under the curve for different pore regions in Fig. 5 was determined through integration. This enabled the quantification of oil mobilization in micropores, mesopores, and macropores at different imbibition stages, with the results presented in Fig. 6. During the early imbibition stage (0–48 h), the oil mobilization degree for micropores, mesopores, and macropores was 30.76%, 33.50%, and 99.91%, respectively. The presence of fractures in the fractured core resulted in the preferential displacement of oil from the fractures and core surface by the oil displacement agent, leading to the highest degree of utilization in macropores during the initial imbibition stage^35,36^. During the middle imbibition stage, mesopores exhibited relatively higher oil mobilization compared to micropores and macropores. Between 48 and 96 h, the oil mobilization degree for micropores, mesopores, and macropores were 10.47%, 29.95%, and 0.086%, respectively. This can be attributed to the smaller pore-throat size and more complex pore structure in micropores, which contain a higher proportion of inaccessible pores. In contrast, mesopores, with relatively simpler structures, continued to contribute significantly to oil mobilization. In the late imbibition stage, simulated oil in micropores showed the highest mobilization extent. Between 96 and 144 h, the mobilization extent of simulated oil in micropores was 15.52%, while that in mesopores was 6.57%, indicating relatively lower mobilization. At this stage, most of the oil in medium pores had already been displaced, and the oil displacement agent continued to penetrate micropores to mobilize the residual oil.

### Characteristics of crude oil flow during depletion production in fracture–matrix models

**Fig. 7:**
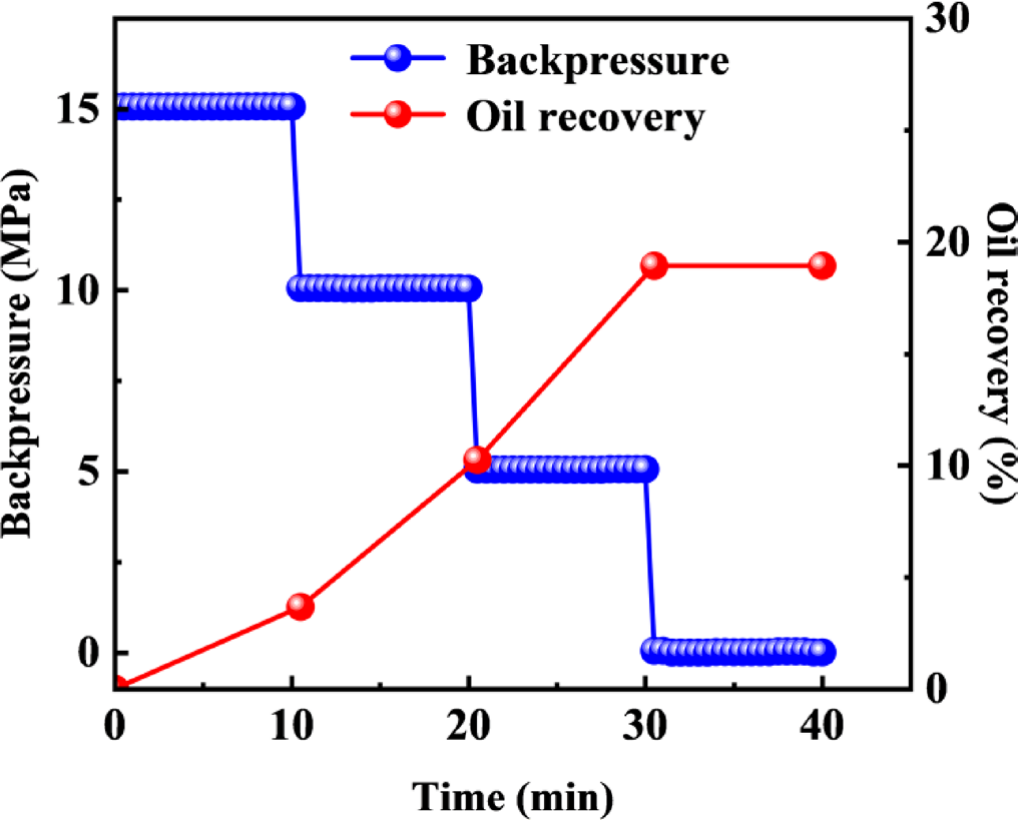
Displacement pressure and oil recovery curve during depletion production.

The results of crude oil depletion production are shown in Fig. 7. When the pressure was reduced from 15 MPa to 10 MPa, the recovery was 3.67%. As the pressure was further reduced to 5 MPa, the recovery increased to 10.25%. When the pressure was lowered to 0 MPa, the recovery reached 18.94%. During production by depressurization, the decrease in reservoir pressure disrupts the original equilibrium: fluids expand elastically due to the pressure drop, while the rock matrix contracts as effective stress increases, leading to pore volume reduction. The combined release of elastic energy from both mechanisms creates a displacement force that overcomes flow resistance, thereby driving oil migration and production.

**Fig. 8:**
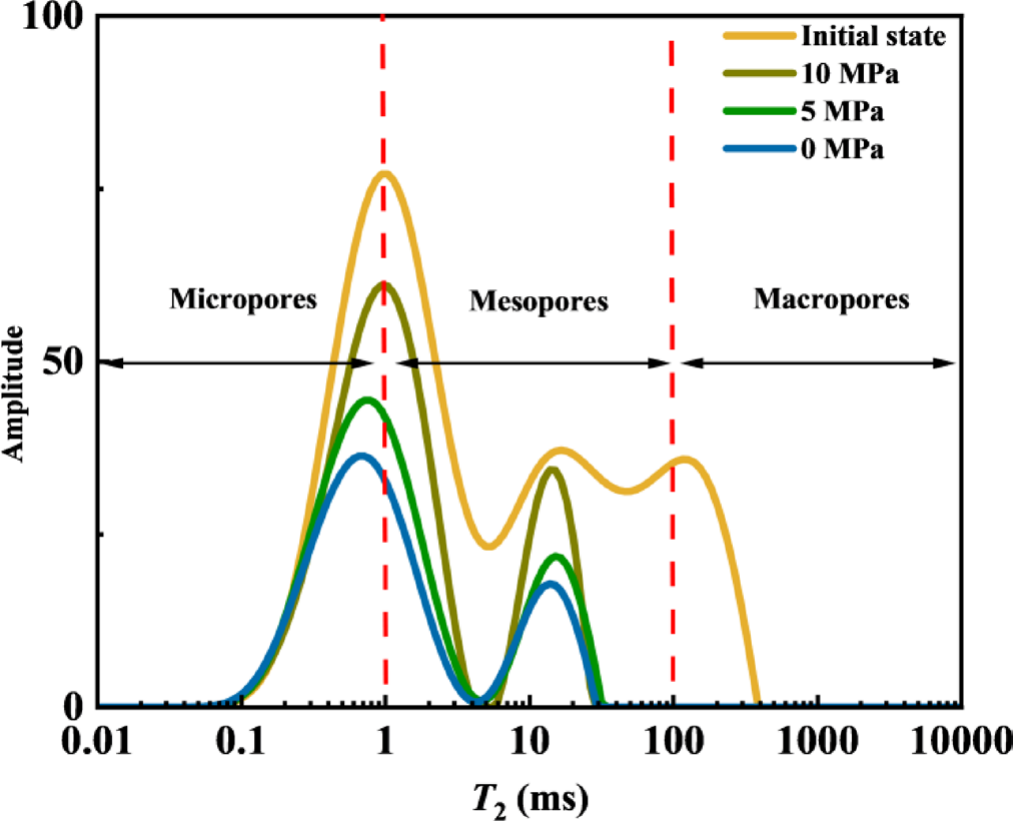
*T*_2_ spectra during depletion production.

**Fig. 9:**
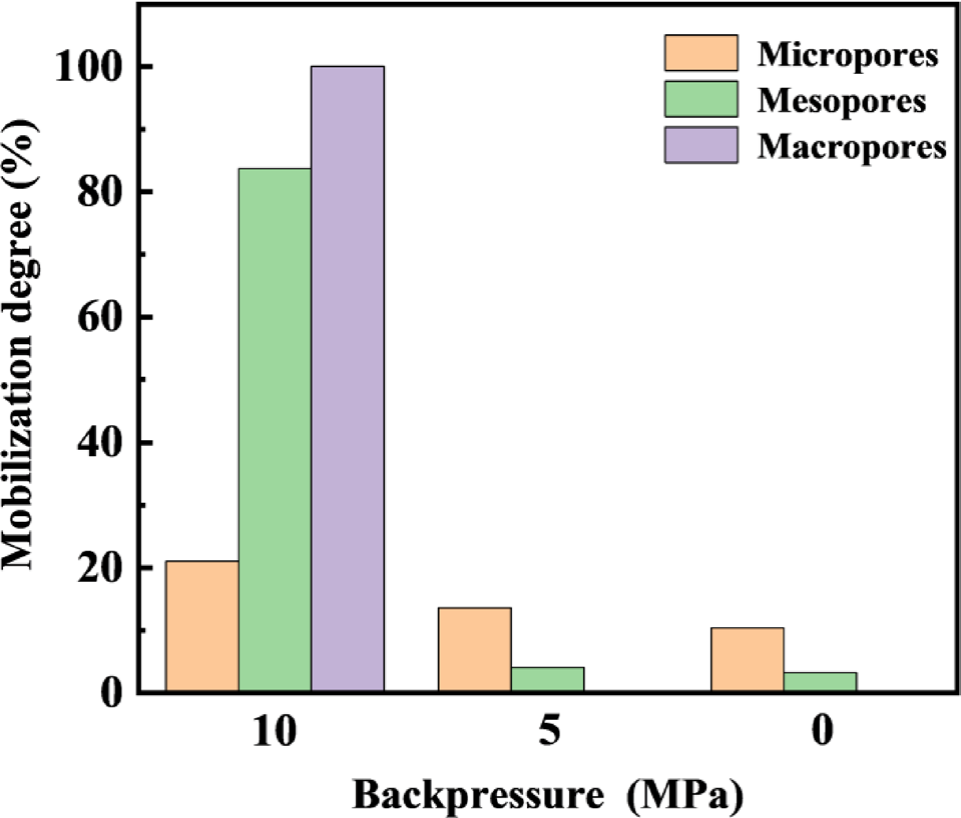
Pore mobilization patterns during depletion production.

As shown in Figs. 8 and 9, the *T*_2_ spectra and pore mobilization patterns during crude oil depletion production indicate that when the pressure was reduced to 10 MPa, a synchronous and significant decline in signals was observed across pores of all scales. Combined with the mobilization patterns presented in Fig. 9, medium and large pores contributed over 60% of the oil mobilization during the early stage, serving as the primary driver of displacement. In the later stage, small pores became the main contributors to oil displacement relative to medium and large pores.

In summary, the oil flow behavior during depletion production can be described as follows: in the early production stage, the fracture system preferentially contributes through medium and large pores, while in the later stage, matrix pores take over as the main contributors, reflecting typical dual-porosity production characteristics.

### Refined optimization of operational parameters based on physically simulated displacement experiments

To optimize the construction parameters for shut-in well stimulation with oil displacement agents, multiple sets of physical simulation displacement experiments were conducted. These experiments systematically investigated the performance differences of various types of oil displacement agents, the displacement efficiency at different concentrations, and the impact of different shut-in durations on imbibition effects. Through comparative analysis of the experimental data, the optimal type of oil displacement agent and its corresponding concentration suitable for the target reservoir conditions were screened. Additionally, the minimum effective soaking time required to achieve the best imbibition displacement effect was determined. This study provides a reliable experimental basis and technical support for the precise optimization of oil displacement agent type, concentration ratio, and soaking process parameters in field pilot tests.

#### Optimization of interfacial tension for oil displacement agents

**Fig. 10:**
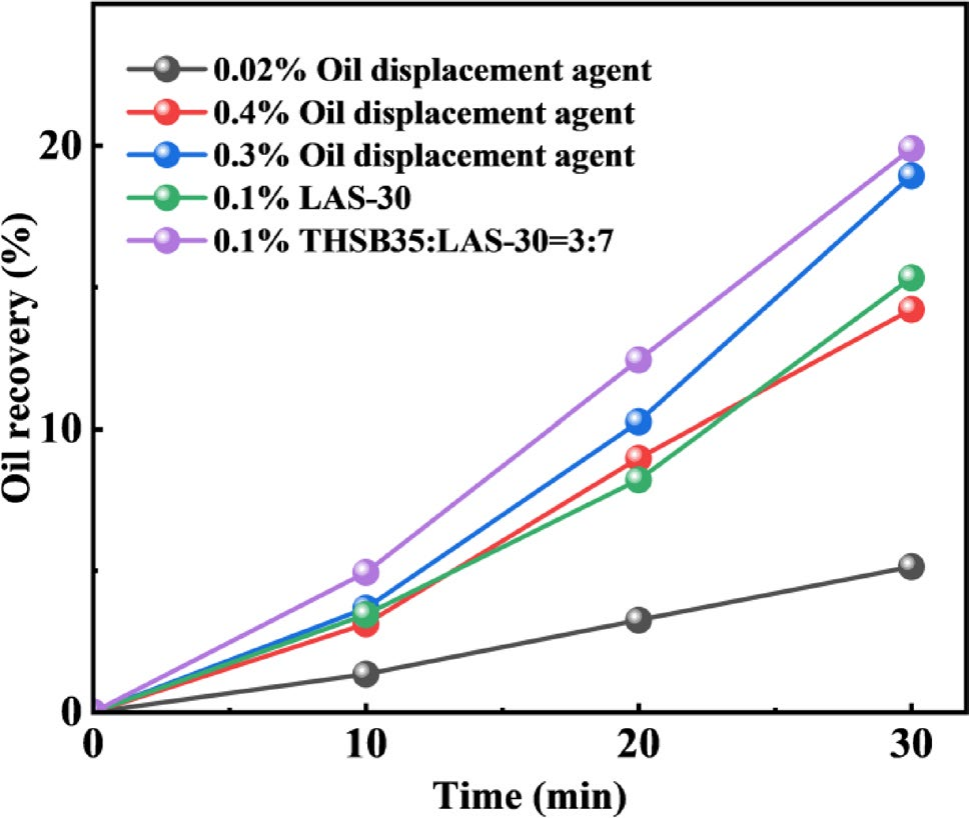
Recovery during depletion production at different interfacial tensions.

As shown in Fig. 10, the recoveries during depletion production at different interfacial tensions indicate that different oil displacement agents exhibit varying displacement performances due to their distinct properties. For the oil displacement agent C-22, when its concentration is 0.02 wt%, the interfacial tension with crude oil exceeds 1 mN/m. When the concentration is increased to 0.3 wt%, the interfacial tension decreases to the order of 10^−2^mN/m. Further increasing the concentration to 0.4 wt% raises the interfacial tension back to the level of 10^−1^ mN/m. At the same concentration of 0.1 wt%, LAS-30 exhibits an interfacial tension with crude oil at the order of 10^−1^ mN/m, while the blended system of LHSB and LAS-30 at a 3:7 ratio can reduce the interfacial tension to the order of 10^−3^ mN/m.

The core mechanism of surfactant flooding technology is to significantly reduce the interfacial tension between oil displacement agent and crude oil, so as to effectively enhance oil recovery. When the interfacial tension is reduced, the capillary resistance required for the migration of crude oil in rock pores can be significantly reduced, making the residual oil more likely to deform and be driven. It can be observed that as the interfacial tension between the oil displacement agent and crude oil decreases to progressively lower levels, the corresponding oil recovery increases to 5.14%, 14.21%, and 18.94%, respectively. When the interfacial tensions of different oil displacement agents with crude oil are within the same order of magnitude, they exhibit relatively similar displacement performance. This is because the oil displacement efficiency is largely controlled by the number of capillaries (which is the ratio of the product of viscosity and flow rate to interfacial tension). The lower the interfacial tension, the larger the number of capillaries, and the more conducive to the start-up of crude oil.

#### Optimization of oil displacement agent concentration

**Fig. 11:**
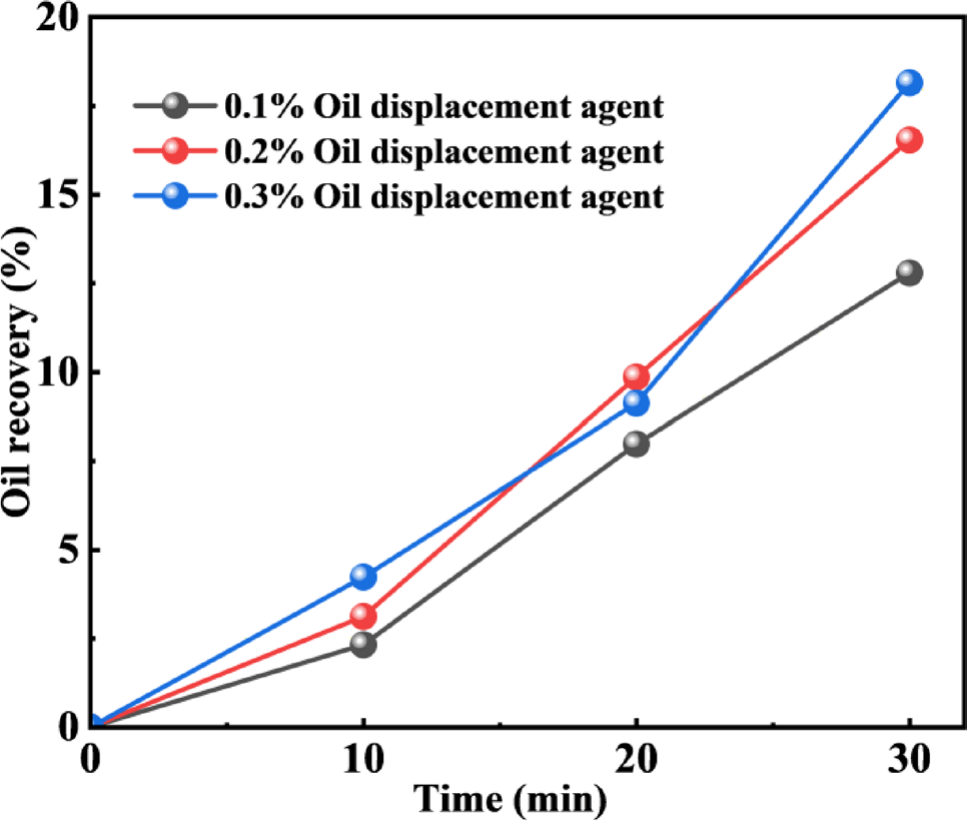
Recovery during depletion production at different oil displacement agents concentrations.

As shown in Fig. 11, the recoveries during depletion production at different oil displacement agent concentrations indicate that after the pressure was reduced to 0 MPa, the recovery was 12.79% at a concentration of 0.1 wt%. As the concentration of the oil displacement agent C-22 increased, the crude oil recovery improved. At a concentration of 0.2 wt%, the recovery was 16.54%. At 0.3 wt% concentration, the recovery reached 18.15%, showing no significant improvement compared to the recovery achieved with the 0.2 wt% concentration solution.

#### Soaking time optimization

Based on the experimental result curves, these three sets of test data clearly demonstrate the influence of shut-in time on crude oil recovery. Overall, a prolonged shut-in period correlates with a higher recovery, as the shut-in process provides sufficient contact time between the injected fluid and the formation crude oil, thereby enhancing the oil recovery efficiency.

**Fig. 12:**
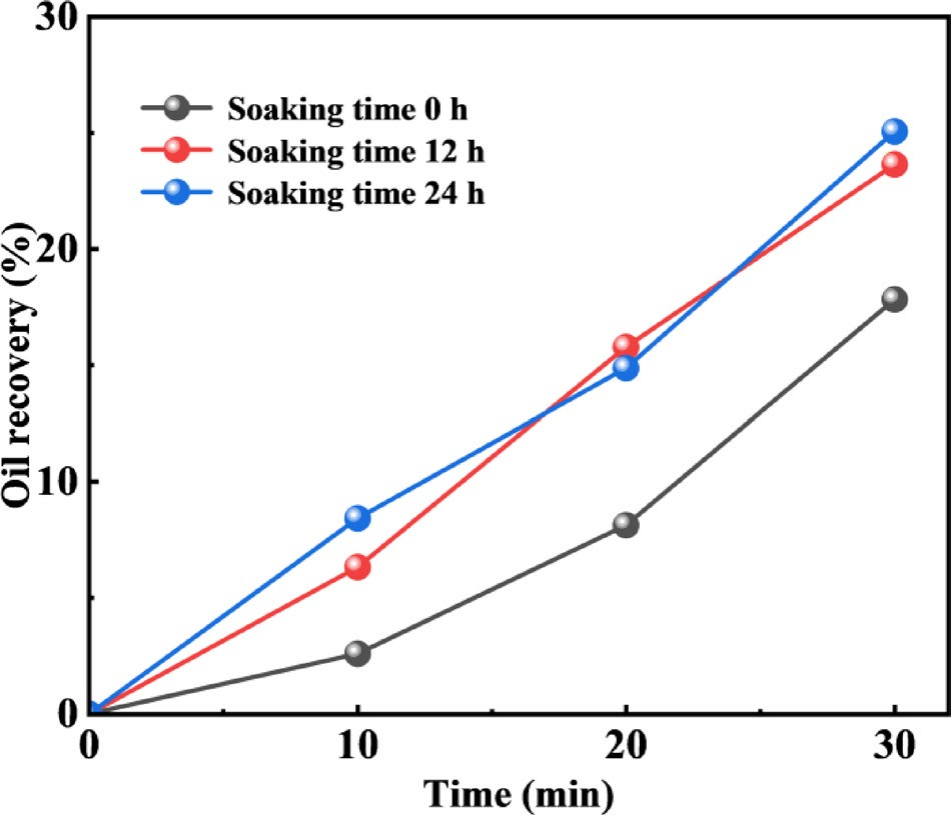
The recovery during depletion production at different soaking time.

As the shut-in time increases, the crude oil recovery shows a corresponding improvement. However, when the shut-in period exceeds 12 h, the enhancement in recovery becomes less significant. This behavior is attributed to the well-connected pore network in sandstone, which facilitates efficient diffusion of the injected fluid during the shut-in period and promotes effective crude oil mobilization. Therefore, extending the soaking time to 12 h results in a substantial increase in recovery, whereas further extension beyond this timeframe yields only marginal additional gains (Fig. 12).

## Conclusions

1. When the concentration of the oil displacement agent C-22 is 0.4 wt%, the interfacial tension between the agent and crude oil is at the order of 10^−1^ mN/m, and the highest spontaneous imbibition recovery achieved is 32.57%. NMR *T*_2_ spectra analysis reveals the mobilization sequence during the imbibition process: in the early stage, oil in large pores and throats is rapidly mobilized, while in the mid to late stages, medium and small pores/throats contribute successively. This indicates that imbibition efficiency is closely related to the mobilization sequence of pore-throat structures.
2. During the production process as pressure was depleted from 15 MPa to 0 MPa, the ultimate recovery reached 18.94%. The mobilization behavior demonstrates a dual-porosity flow mechanism: early production is primarily dominated by the fracture system (medium and large pores/throats), while later production relies on supplementary contribution from matrix pores. This pattern indicates that effectively mobilizing the crude oil within the matrix is critical for enhancing the performance of depletion-based production.
3. Based on the physical simulation experiments conducted in this work, an optimal parameter combination for maximizing oil recovery in tight sandstone reservoirs has been identified. Specifically, the interfacial tension should be controlled at the order of 10^−2^ mN/m, the optimal concentration of C-22 is 0.3 wt%, and the soaking time is 12 h. This parameter set ensures effective displacement while promoting coordinated recovery from both fractures and matrix pores.

## Data Availability

The datasets generated and/or analysed during the current study are not publicly available due to the proprietary nature of the oil displacement agent and its supporting data, which are currently in the initial stage of field application. Public release at this critical phase could compromise commercial interests and ongoing technology transfer agreements. The data are available from the corresponding author on reasonable request, subject to a confidentiality agreement.
